# Folate, Cobalamin, Cysteine, Homocysteine, and Arsenic Metabolism among Children in Bangladesh

**DOI:** 10.1289/ehp.0800164

**Published:** 2009-01-15

**Authors:** Megan N. Hall, Xinhua Liu, Vesna Slavkovich, Vesna Ilievski, J. Richard Pilsner, Shafiul Alam, Pam Factor-Litvak, Joseph H. Graziano, Mary V. Gamble

**Affiliations:** 1 Department of Epidemiology; 2 Department of Biostatistics and; 3 Department of Environmental Health Sciences, Columbia University, New York, New York, USA;; 4 Department of Epidemiology, University of Michigan, Ann Arbor, Michigan, USA;; 5 Columbia University Arsenic Project, Bangladesh, Dhaka, Bangladesh

**Keywords:** arsenic, Bangladesh, children, cobalamin, creatinine, cysteine, dimethylarsinic acid, folate, homocysteine, monomethylarsonic acid, one-carbon metabolism

## Abstract

**Background:**

Approximately 35 million people in Bangladesh are chronically exposed to inorganic arsenic (InAs) in drinking water. Methylation of InAs to monomethylarsonic (MMA) and dimethylarsinic acids (DMA) relies on folate-dependent one-carbon metabolism and facilitates urinary arsenic (uAs) elimination.

**Objectives:**

We examined the relationships between folate, cobalamin, cysteine, total homocysteine (tHcys), and uAs metabolites in a sample of 6-year-old Bangladeshi children (*n* = 165).

**Methods:**

Children provided blood samples for measurement of tHcys, folate, cobalamin, and cysteine, and urine specimens for the measurement of total uAs and As metabolites.

**Results:**

Consistent with our studies in adults, mean tHcys concentrations (7.9 μmol/L) were higher than those reported among children of similar ages in other populations. Nineteen percent of the children had plasma folate concentrations < 9.0 nmol/L. The proportion of total uAs excreted as InAs (%InAs) was inversely correlated with folate (*r* = −0.20, *p* = 0.01) and cysteine (*r* = −0.23, *p* = 0.003), whereas the correlations between %DMA and both folate (*r* = 0.12, *p =* 0.14) and cysteine (*r* = 0.11, *p* = 0.15) were positive. Homocysteine was inversely correlated (*r* = −0.27, *p* = 0.009) with %MMA in males, and the correlation with %DMA was positive (*r* = 0.13, *p* = 0.10).

**Conclusions:**

These findings suggest that, similar to adults, folate and cysteine facilitate As methylation in children. However, the inverse correlation between tHcys and %MMA, and positive correlation with %DMA, are both opposite to our previous findings in adults. We propose that upregulation of one-carbon metabolism, presumably necessary to meet the considerable demands for DNA and protein biosynthesis during periods of rapid growth, results in both increased tHcys biosynthesis and increased As methylation.

Arsenic is a class I human carcinogen [International Agency for Research on Cancer (IARC) 2004] to which approximately 140 million people worldwide, including 35 million in Bangladesh alone (Kinniburgh and Smedley 2001), are chronically exposed through contaminated drinking water. In our previous survey of water As concentrations of all tube wells within our study region in Araihazar, Bangladesh (Van Geen et al. 2002), 72% of the wells exceeded both the U.S. Environmental Protection Agency (U.S. EPA 2001) and the World Health Organization (WHO 2004) limits of 10 μg/L, and 52% exceeded the Bangladesh standard of 50 μg/L. Chronic exposure to As has been associated with increased risk of several cancers, including lung, bladder, liver, and skin (California Environmental Protection Agency 2004; Navarro Silvera and Rohan 2007), as well as ischemic heart disease, neurologic sequelae (California Environmental Protection Agency 2004), and deficits in intelligence in children (Wasserman et al. 2004).

As is metabolized in humans via reduction and methylation reactions, with *S*-adenosylmethionine (SAM) serving as the methyl donor (Marafante and Vahter 1984). Methylation of inorganic As (InAs) to monomethylarsonic (MMA) and dimethylarsinic acids (DMA) occurs enzymatically via one-carbon metabolism (Figure 1) and facilitates urinary As (uAs) elimination (Vahter and Marafante 1987). Methylation of As has generally been considered a detoxification pathway; studies show that a higher proportion of DMA in urine is associated with decreased risk of skin lesions (Ahsan et al. 2007), skin (Chen et al. 2003a) and bladder cancers (Chen et al. 2003b; Huang et al. 2008), and peripheral vascular disease (Tseng et al. 2005). There is, however, accumulating evidence that the trivalent methylated As intermediate, MMA^III^, may be the most toxic As species (Styblo et al. 2002; Wang et al. 2007).

Individuals show substantial variation in As methylation capacity, which may be partly attributable to availability of vitamins required for one-carbon metabolism [folate, vitamin B_12_ (cobalamin), and vitamin B_6_] (Figure 1) as well as intake of nutrients that are not required per se but that contribute to the availability of methyl groups, such as protein, betaine, and choline. In animal models, folate deficiency decreases uAs excretion (Spiegelstein et al. 2003), and dietary methyl donor deficiency increases As retention in tissues (Vahter and Marafante 1987). In Bangladeshi adults, plasma folate was inversely associated with %MMA and positively associated with %DMA in urine (Gamble et al. 2005b). Further, in our randomized controlled trial, folic acid supplementation to folate-deficient adults was associated with increased %DMA in urine (Gamble et al. 2006) and a statistically significant 14% reduction in total blood As (Gamble et al. 2007), primarily due to a decline in blood MMA. These findings emphasize the importance of adequate folate for the synthesis and relatively rapid elimination of As as DMA.

We have also previously observed that urinary creatinine—an analyte traditionally used to adjust for hydration status (and a catabolite of creatine)—is a strong predictor of As methylation (Ahsan et al. 2007; Gamble and Liu 2005; Gamble et al. 2005b, 2006; Hall et al. 2007) and that participants with lower urinary creatinine are at increased risk for As-induced skin lesions (Pilsner et al. 2008). Urinary creatinine is influenced by dietary intake of creatine (derived from meat), which downregulates endogenous creatine biosynthesis. Because creatine biosynthesis is the major consumer of methyl groups (Mudd and Poole 1975; Stead et al. 2006), downregulation by dietary sources may lower total homocysteine (tHcys) (Korzun 2004; Stead et al. 2001; Taes et al. 2003) and thereby increase the pool of methyl groups for methylation of As and other substrates.

Few studies have examined As methylation in children. Concha et al. (1998) reported higher %InAs and lower %DMA in urine among children in northern Argentina compared with women, suggesting that children may have reduced capacity to methylate As. However, Chowdhury et al. (2003) reported that children in Bangladesh had lower %InAs and %MMA and higher %DMA than adults. Both our own work in Bangladeshi children (Wasserman et al. 2004) and the results of a recent study conducted in China are not supportive of the finding by Concha et al. (1998). For example, Sun and colleagues (2007) reported that children had lower %MMA and higher %DMA in urine than adults when exposed to the same concentration of As in drinking water. Meza et al. (2005) reported that three single nucleotide polymorphisms in the *AS3MT* gene were associated with increased As methylation in children but not in adults (Meza et al. 2005). Together, these studies suggest that children have a greater capacity to methylate As than adults.

To our knowledge, there are no published reports pertaining to nutritional influences on As metabolism in children. Given that childhood may represent a critical period for exposure to As with regard to risk for long-term adverse health outcomes (Smith et al. 2006; Wasserman et al. 2007; Yuan et al. 2007), it is important to identify potentially modifiable risk factors that may favorably influence As metabolism and elimination. In this study, we examined the prevalence of folate deficiency and hyperhomocysteinemia and the relationships between folate, cobalamin, cysteine, tHcys, and As metabolism in 6-year-old Bangladeshi children.

## Methods

### Overview

This work is part of the Nutritional Influences on Arsenic Toxicity (NIAT) study (Gamble et al. 2005a, 2005b, 2006) in collaboration with a larger, multidisciplinary program [The Columbia University Superfund Basic Research Program (CU-SBRP)]. The main health project within the CU-SBRP is the Health Effects of Arsenic Longitudinal Study (HEALS) (Ahsan et al. 2006), a prospective cohort study of > 12,000 married men and women living in Araihazar, Bangladesh, who are followed up at 2-year intervals. The study region and recruitment of HEALS participants have been previously described (Ahsan et al. 2006). Araihazar was chosen because it has a wide range of As concentrations in drinking water and is within a reasonable commuting distance from Dhaka.

### Study participants and procedure

We identified a sample of 6-year-old children of HEALS participants who were available at the time of a preliminary home visit and were willing to provide biological samples. The current study sample is a subset of 165 of 301 children selected at random for our previous study of water As exposure and intellectual function who were willing to provide both blood and urine samples (Wasserman et al. 2007). This subset of 165 children did not differ from the 136 who gave only a urine sample with regard to total uAs or percent uAs metabolites. However, the 136 children who did not give blood samples weighed 0.5 kg less, were 1.5 cm shorter, had head circumferences that were 0.3 cm smaller, and had higher water As (131 vs. 111 μg/L, respectively) than those who did donate blood. The research protocol was approved by the Bangladesh Medical Research Council and the Columbia University Medical Center Institutional Review Board. Informed oral parental consent and child assent were obtained by field staff physicians.

Height, weight, and head circumference were measured, and blood and urine samples were obtained during a visit to our field clinic in Araihazar by the children and their mothers. Blood samples were collected into EDTA-containing tubes, immediately placed in IsoRack Cool packs (Brinkmann Instruments, Westbury, NY, USA), and centrifuged within 1 hr at 4°C to separate plasma and cells. Plasma was then stored at −80°C and shipped frozen on dry ice to Columbia University for analysis. Spot urine samples were collected in 50-mL acid-washed tubes, frozen at −20°C, and also shipped on dry ice. Family demographics were obtained from the original baseline interview of parents in the HEALS cohort study. Well water As concentrations were measured as part of the survey of all wells in the study region (Van Geen et al. 2002), and mothers were asked to identify the primary source of the child’s drinking water.

### Measurement of plasma nutrients

We analyzed plasma folate and cobalamin by radioimmunoassay (Quantaphase II; Bio-Rad Laboratories, Richmond, CA, USA) as previously described (Gamble et al. 2005a; Pfeiffer et al. 2005). The within-day coefficient of variation (CV) was 3% for folate and 4% for cobalamin. The between-day CV was 5% for folate and 11% for cobalamin. We used high-performance liquid chromatography (HPLC) with fluorescence detection (Pfeiffer et al. 1999) to measure plasma tHcys and cysteine concentrations as described previously (Gamble et al. 2005a). The within-day and between-day CVs were 3% and 6% for tHcys and 5% and 8% for cysteine, respectively.

### Well water As

We analyzed well water samples for total As by graphite furnace atomic absorption (GFAA) with a detection limit of 5 μg/L. Samples found to have a concentration < 5 μg/L were reanalyzed by inductively coupled plasma-mass spectrometry (ICP-MS) for which the detection limit is 0.1 μg/L (Cheng et al. 2004).

### Total uAs and creatinine

Total uAs was measured in the Columbia University Trace Metals Core Laboratory, as described previously (Nixon et al. 1991), by GFAA spectrometry using the Analyst 600 graphite furnace system (PerkinElmer, Shelton, CT, USA). Our laboratory is part of a quality control program organized by P. Weber at the Quebec Toxicology Center in Quebec, Canada. Intraclass correlation coefficients between our laboratory’s values and samples calibrated at the Quebec laboratory were 0.99. Urinary creatinine concentrations were analyzed using a method based on the Jaffe reaction (Slot 1965).

### uAs metabolites

As metabolites were speciated using HPLC separation of arsenobetaine (AsB), arsenocholine (AsC), arsenate (InAs^V^), arsenite (InAs^III^), MMA, and DMA followed by detection using ICP-MS. After subtracting AsC and AsB from the total, we calculated the percentages of InAs (InAs^III^ + InAs^V^), MMA (MMA^III^ + MMA^V^), and DMA^V^.

### Statistical analysis

Plasma folate, cobalamin, tHcys, and cysteine, and uAs metabolites were not normally distributed. We therefore used nonparametric tests where appropriate and log transformations to create approximately normal distributions before using linear regression.

We calculated descriptive statistics for general characteristics of the participants separately by sex and tested for sex differences using the Wilcoxon rank-sum test for continuous variables and the chi-square test for categorical variables. We analyzed correlations between plasma nutrients, between nutrients and covariates, as well as between nutrients and percent uAs metabolites using Spearman’s correlation coefficients. We used linear regression to examine predictors of %InAs, %MMA, and %DMA. Potential confounders considered for inclusion in the regression models were age, sex, urinary creatinine, albumin, and body mass index (BMI). Although the range for age was narrow (5.75–6.5 years), we included it in the regression models because it was associated with folate and tHcys as well as %MMA. All analyses were performed using SAS version 9.1 (SAS Institute Inc., Cary, NC, USA).

## Results

**Figure 1:**
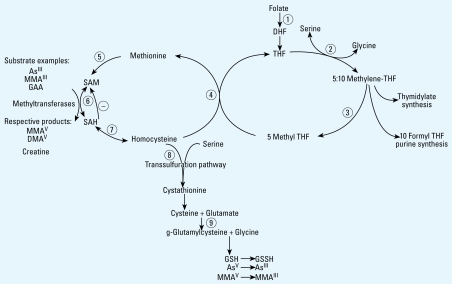
Overview of one-carbon metabolism. (*1*) Dietary folates are reduced to dihydrofolate (DHF) and tetrahydrofolate (THF) by dihydrofolate reductase. (*2*) Serine hydroxymethyl-transferase transfers 1-carbon units from serine to THF, forming 5,10-methylene-THF and glycine. (*3*) 5,10-methyl THF reductase can reduce 5,10-methylene-THF to 5-methyl-THF. (*4*) In a reaction catalyzed by methionine synthetase, the methyl group of 5-methyl-THF is transferred to homocysteine, generating methionine and THF. (*5*) Methionine adenosyltransferase activates methionine to form SAM. (*6*) SAM is a methyl donor for a variety of acceptors, including guanidinoacetate (GAA, precursor to creatine), DNA, and As, in reactions that involve a number of methyltransferases. (*7*) The by-product of these methylation reactions, *S*-adenosylhomocysteine (SAH), is hydrolyzed to regenerate Hcys. (*8*) Hcys is either used to regenerate methionine or is directed to the transsulfuration pathway through which it is ultimately catabolized. (*9*) The transsulfuration pathway is also responsible for glutathione (GSH) biosynthesis.

The general characteristics of the study sample by sex are presented in Table 1. As expected, males were taller and heavier than females and also had a larger head circumference. To our knowledge, there are no standards for BMI or plasma folate, cobalamin, and homocysteine that are specific to the Bangladeshi population or to Asian populations in general. We therefore used the best available cutoffs for estimating low BMI for age, folate and cobalamin deficiency, and high homocysteine in other populations, fully appreciating the limitations of this approach. Based on age-specific growth charts from the Centers for Disease Control (CDC) (Kuczmarski et al. 2002), 52% of females and 49% of males had a BMI below the 5th percentile. Using WHO growth standards (de Onis et al. 2007), these percentages were 38.4% for females and 33.7% for males. Almost all of the children had albumin levels < 3.8 g/dL. Using ird National Health and Nutrition Examination Survey (NHANES III) data for 6- to 11-year-old U.S. children (Must et al. 2003), males had a higher prevalence of high homocysteine (78% > 95th percentile, i.e., > 7.0 μmol tHcys/L) than did females (64% > 7.0 μmol/L). In the absence of published reference values for children, we used published adult reference values for marginal plasma folate (< 9 nmol/L) (Christenson et al. 1985); 18% of females and 21% of males were classified as having marginal folate nutritional status [see Supplemental Material, Figure 1 (available online at http://www.ehponline.org/members/2009/0800164/suppl.pdf)]. Using the CDC adult cutoff of 6.8 nmol/L (Pfeiffer et al. 2005), 4.1 % of females and 3.3 % of males were classified as having folate deficiency. Similarly, using adult reference values for cobalamin (Christenson et al. 1985), 7% of females and 5% of males had plasma levels < 151 pmol/L, indicative of deficiency.

As expected, both plasma folate and cobalamin were inversely correlated with tHcys (*r* = −0.21, *p* = 0.008 and *r* = −0.14, *p* = 0.07, respectively). After adjustment for age and sex, plasma folate explained only 3.5% and cobalamin explained 2.3% of the variation in plasma tHcys. tHcys and cysteine were positively correlated (*r* = 0.43, *p* < 0.0001); this association was stronger among females (*r* = 0.64, *p* < 0.0001) than males (*r* = 0.23, *p* = 0.03) and the sex difference was statistically significant (*p* < 0.01) [see Supplemental Material, Table 1 (available online at http://www.ehponline.org/members/2009/0800164/suppl.pdf)].

Water As concentrations ranged from 0.1 to 864 μg/L; 56% of the wells that served as the primary source of drinking water for these children had water As concentrations above the Bangladesh standard of 50 μg/L, whereas 79% were above the WHO standard of 10 μg/L. Urinary creatinine concentrations were similar for males and females; both total uAs and total uAs per gram creatinine were significantly higher among males (Table 1).

Consistent with our previous findings in adults, plasma folate was inversely associated with both total uAs (*r* = −0.22, *p* = 0.004) and uAs per gram creatinine (*r* = −0.31, *p* < 0.0001). Plasma levels of cobalamin, tHcys, and cysteine were not significantly associated with either total uAs or total uAs per gram creatinine (Table 2). Overall, there were no statistically significant correlations between the plasma measures and urinary creatinine (data not shown). However, there was a positive association between plasma folate and urinary creatinine among females (*r* = 0.22, *p* = 0.06).

**Figure 2:**
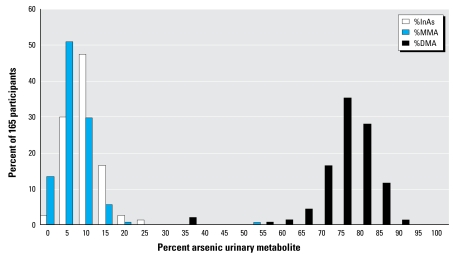
Frequency distribution for As metabolites.

Figure 2 shows the frequency distributions of As metabolites in urine, expressed as percent of total uAs. Mean levels (± SD) of %InAs (12.4 ± 5.3), %MMA (9.0 ± 3.6), and %DMA (78.7 ± 6.3) did not differ significantly between males and females (Table 1). Both plasma folate and cysteine were inversely associated with %InAs, and for cysteine the association was stronger among males than females (Table 2). Completely contrary to our previous findings in adults, plasma tHcys was inversely correlated with %MMA; this association was limited to males. The correlations between plasma levels of tHcys, cysteine, and folate with %DMA were all positive but were not statistically significant (Table 2). As we previously observed in adults, urinary creatinine was inversely correlated with %InAs (*r* = −0.31, *p* < 0.0001) and positively correlated with %DMA (*r* = 0.22, *p* < 0.0001) (Table 2).

Compared with our previous cross-sectional study of 293 adults, children from both the current study and from our previous study of 201 10-year-old children (Wasserman et al. 2004) appeared to have lower urinary %InAs and %MMA and higher %DMA (Table 3). These differences persisted after adjustment for water As and plasma folate (data not shown).

The results from linear regression analyses for the associations between plasma nutrients (categorized by quartiles) and uAs metabolites, after adjustment for age, sex, albumin, and total uAs, showed the same patterns of association as did the Spearman’s correlation coefficients. Increasing plasma folate or cysteine was associated with decreased %InAs, although the effect estimate was not statistically significant for folate [b for highest versus lowest quartile was −0.13 (*p* = 0.10) for folate and −0.18 (*p* = 0.02) for cysteine]. There were positive but not statistically significant associations between %DMA and both plasma folate (b = 0.03, *p* = 0.11 for highest vs. lowest quartile) and cysteine (b = 0.03, *p* = 0.21 for highest versus lowest quartile). Increasing plasma tHcys was associated with decreased %MMA (b = −0.18, *p* = 0.05), and this association was of borderline statistical significance. As we previously observed in adults (Gamble et al. 2005b), additional adjustment for urinary creatinine generally resulted in attenuation of associations between plasma variables and uAs metabolites. To further explore sex differences in the association between tHcys and %MMA, we fit separate linear regression models by sex. Among males, the parameter estimate for the difference in mean %MMA between the highest and lowest quartiles of tHcys after adjustment for age, albumin, and total uAs was −0.31 (*p* = 0.02) and with further adjustment for urinary creatinine was −0.29 (*p* = 0.03). For females, the parameter estimate was −0.04 (*p* = 0.78). This sex difference did not reach statistical significance of 0.05 based on a Wald test.

## Discussion

In this cross-sectional study of 6-year-old Bangladeshi children (*n* = 165), the associations between plasma folate and As metabolites observed were similar to those in our study of adults (Gamble et al. 2006); folate was inversely correlated with %InAs and positively correlated with %DMA, although the latter did not achieve statistical significance. These findings suggests that folate is required for both the first and second As methylation steps and are in line with the well-known role of folate as a methyl donor involved in the generation of SAM. We did not observe an association between folate and %MMA in children, whereas in adults there was a statistically significant inverse association. Consistent with our previous results (Gamble et al. 2006), we did not find associations between plasma cobalamin and any of the uAs metabolites, although the ability to detect an association in our previous study was limited by the exclusion of cobalamin deficient participants. Our relatively small sample size and the small number of children with cobalamin deficiency may have constrained our ability to detect an association in this study.

Plasma cysteine showed a statistically significant inverse association with %InAs and was positively associated with %MMA and %DMA, suggesting that cysteine may be involved in As reduction, thus facilitating both the first and second As methylation steps. Cysteine is an amino acid that can be produced from the catabolism of Hcys and is an intermediate in the synthesis of glutatione (Figure 1). Cysteine is involved in redox cycling (Jones et al. 2004) and can reduce As^V^ to As^III^ *in vitro* (Celkova et al. 1996), a necessary step before methylation. Although our findings suggest that the reported ability of cysteine to reduce As *in vitro* may also be relevant in humans, it is equally possible that the association is secondary to the role of cysteine in glutathione biosynthesis; glutathione is known to be capable of providing reducing equivalents for this reaction.

Plasma tHcys was significantly inversely associated with %MMA in males, and its estimated correlation with %DMA was positive, although not statistically significant, among both males and females. These associations are opposite of those in adults. Although additional studies will be required to clarify the mechanism underlying these observations, the findings raise the question of whether there may be differences in the fundamental regulation of one-carbon metabolism between children and adults. Theoretically, one would expect overall upregulation of one-carbon metabolism during periods of rapid growth to meet the high demands for protein and DNA synthesis. In rats and rabbits, glycine-*N*-methyltransferase (GNMT) activity levels are very low at birth and increase continuously with age (Heady and Kerr 1975). Because GNMT, which catalyzes the nonessential methylation of glycine to sarcosine, competes for SAM and generates SAH, lower activity during periods of rapid growth may be one mechanism whereby increased requirements for SAM during growth and development are achieved. Whether similar developmental changes occur in humans is unknown.

The mean plasma tHcys concentrations (7.9 μmol/L) in our study are generally higher than those reported among children of similar ages in other populations (Bates et al. 2002; Must et al. 2003; Papandreou et al. 2006). In 6- to 11-year-old participants in NHANES III (1988–1994, prefolate fortification), mean plasma tHcys concentrations were 5.2 μmol/L among boys and 5.3 μmol/L among girls (Must et al. 2003). Geometric mean plasma tHcys concentrations were somewhat higher in 6- to 9-year-old Greek children (6.5 μmol/L) (Papandreou et al. 2006). Differences in folate status may partially explain the higher tHcys concentrations in our population; in 4- to 11-year-old participants in NHANES III, mean plasma folate levels measured were 19.9 nmol/L (Pfeiffer et al. 2007), substantially higher than the mean concentrations observed in this study (12.8 nmol/L). However, it is likely that other factors—for example, genetics—may contribute to these considerable population differences in tHcys concentrations, given that plasma folate explained only a small fraction of the variability in tHcys concentrations.

In our previous study of Bangladeshi adults (Gamble et al. 2005a), we observed a pronounced sex difference in the prevalence of hyperhomocysteinemia (males > females); among children, the sex difference was of borderline statistical significance. Must et al. (2003) also reported a sex divergence in tHcys concentrations that began at approximately 10 years of age and continued through adolescence. This sex difference is often attributed to steroid hormones, an explanation that is not relevant for this sample of young children not yet approaching puberty.

As expected, average tHcys concentrations were lower among children compared with our study of adults (adults: females, 9.5 μmol/L and males, 15.3 μmol/L; children: females, 7.7 μmol/L and males, 8.0 μmol/L). However, using NHANES III cutoff values for each age category, we observed an overall higher prevalence of high homocysteine (78% and 64% > 7 μmol/L for males and females, respectively) than in our study of adults, in which the prevalence was 26% among females (> 10.4 μmol/L) and 63% among males (> 11.4 μmol/L) (Gamble et al. 2005a). Although a high prevalence of hyperhomocysteinemia among Asian adults has been previously reported (Carmel et al. 2002; Chambers et al. 2000), this finding in children is somewhat surprising, given that the children have a lower prevalence of folate and cobalamin deficiencies. The correlations between plasma folate and tHcys as well as between cobalamin and tHcys were also weaker in this study than in the adults (Gamble et al. 2005a), and plasma folate accounted for a smaller portion of the variability of tHcys (3.5% vs. 15% for children and adults, respectively). Perhaps in children, homocysteine biosynthesis makes a relatively greater contribution to tHcys concentrations than its effective removal via remethylation compared with adults.

Our current findings suggest that children have a lower mean urinary %InAs and %MMA and higher mean %DMA than do adults, independent of water As and plasma folate levels, suggesting an overall higher As methylation capacity. This is in agreement with most other studies comparing adults and children and is consistent with our conjecture that one-carbon metabolism may be generally upregulated during periods of growth. Behaviors prevalent in Bangladeshi adults that are known to be associated with reduced As methylation, such as cigarette smoking and betel nut use, may also partially explain this finding.

The expression of uAs metabolites as percentages limits the interpretation of these findings, because the relative level of each metabolite is influenced by that of the others. Likewise, the lack of data on blood As metabolites is a limitation, because the relative distribution of As metabolites in urine does not closely reflect those in blood; MMA makes up a substantially greater proportion of total blood As than urine As both in adults (Gamble et al. 2007) and in children (Hall et al. 2007). However, urine As metabolites are still useful for indicating overall patterns of association.

In this study we also did not examine the influence of genetic factors on the associations between plasma nutrients and As metabolites. Polymorphisms in enzymes involved in folate and homocysteine metabolism as well as *AS3MT* may contribute to individual variability in As metabolism in this population and should be investigated in future studies. An additional limitation is that the low albumin levels observed here may be attributable to an acute-phase response associated with undiagnosed infections and may not accurately reflect the prevalence of protein deficiency among children in this region; infections are common among children in the region. However, based on the information on height, weight, and BMI for age provided in Table 1, a substantial portion (about one-third) of our study participants appear to be malnourished. Also, age was self-reported by the parents, and birth certificates were not available to confirm ages. Any measurement error in the ascertainment of age could lead to inadequate control for potential confounding by age. Other limitations include the lack of a measure of plasma choline or betaine.

In conclusion, the results of this cross-sectional study suggest that folate and cysteine facilitate As methylation in children; these findings are similar to those of adults. However, the observed negative association between tHcys and %MMA and the positive association with %DMA are opposite those of adults. Additional studies will be required to clarify the mechanism underlying these observations. The unusually high prevalence of high homocysteine concentrations among 6-year-old Bangladeshi children, despite a lower prevalence of folate deficiency than among adults from the same geographic area, was likewise unanticipated. Determinants of tHcys and the potential long-term health implications of high tHcys in children are not well characterized and warrant further study. Finally, these results suggest an overall high As methylation capacity in children compared with adults. Collectively, these observations underscore the need for further study of the regulation of one-carbon metabolism and its influence on As methylation and toxicity during periods of rapid growth, time periods that may be critical with regard to long-term health outcomes.

## Figures and Tables

**Table 1:** General characteristics, plasma measurements, and levels of As variables.

	Female (*n* = 73)		Male (*n* = 92)		
Characteristic	Mean ± SD	Median (range)	Mean ± SD	Median (range)	*p*-Value^a^
Age (years)	6.1 ± 0.2	6.2 (5.8–6.5)	6.1 ± 0.2	6.1 (5.8–6.3)	0.59
Height (cm)	108.9 ± 5.7	109.4 (97.0–122.2)	109.4 ± 4.8	109.7 (98.2–120.5)	0.54
Weight (kg)	15.9 ± 2.0	15.7 (11.8–20.8)	16.5 ± 1.8	16.5 (12.2–21.5)	0.03
BMI (kg/m^2^)	13.4 ± 1.1	13.4 (9.4–15.9)	13.8 ± 1.1	13.7 (10.2–16.2)	0.03
BMI < 3rd percentile (%)^b^	28.8		27.2		0.69
Head circumference (cm)	47.7 ± 1.2	47.8 (44.8–50.5)	49.1 ± 1.3	49.0 (45.4–52.6)	< 0.0001
Height for age < 3rd percentile (%)	30.1		27.2		
Weight for age < 3rd percentile (%)	39.7		35.9		
Albumin (g/dL)	2.6 ± 0.4	2.5 (1.8–4.3)	2.6 ± 0.4	2.4 (1.7–4.3)	0.50
Albumin < 3.8 g/dL (%)	97.3		97.8		1.0
Type of housing (%)					
Thatched	8.2		15.2		0.21
Corrugated tin	83.6		75.0		
Other	8.2		9.8		
Plasma tHcys (μmol/L)	7.7 ± 1.5	7.5 (4.8–13.7)	8.0 ± 1.4	7.9 (5.3–12.7)	0.15
High homocysteine (%)^c^	64.4		78.3		0.05
Plasma folate (nmol/L)	13.3 ± 4.8	12.9 (6.2–32.9)	12.4 ± 5.0	11.3 (4.7–38.1)	0.12
Plasma folate < 9.0 nmol/L (%)	17.8		20.7		0.65
Plasma cobalamin (pmol/L)	338.5 ± 140.6	315.3 (86.3–731.6)	314.9 ± 111.8	318.6 (124.3–678.3)	0.41
Plasma cobalamin < 151 pmol/L (%)	6.9		5.4		0.75
Plasma cysteine (μmol/L)	152.2 ± 26.2	153.0 (77.0–219.0)	152.9 ± 20.6	151.0 (105.0–202.0)	0.93
Water As (μg/L)	97.6 ± 119.7^a^		121.4 ± 140.4		0.24
Urinary creatinine (mg/dL)	38.9 ± 27.1		36.9 ± 29.1		0.39
uAs (μg/L)	85.0 ± 69.7		116.1 ± 112.1		0.04
uAs/g creatinine	268.9 ± 263.7		359.0 ± 254.0		0.007
%InAs	12.6 ± 6.8		12.2 ± 3.9		0.49
%MMA	9.0 ± 3.3		8.9 ± 3.8		0.57
%DMA	78.4 ± 7.1		78.8 ± 5.5		0.92

a For test of difference by sex, based on Wilcoxon’s rank-sum test for continuous variables and chi-square or Fisher’s exact test for categorical variables.

b Fifth percentile based on sex-specific BMI for age, WHO growth standards: 13.72 for males and 13.46 for females (de Onis et al. 2007).

c Defined as tHcys > 7.0 μmol/L based on 95th percentile in 6- to 11-year-olds from NHANES III data (Must et al. 2003).

**Table 2:** Spearman’s correlation coefficients between folate, cobalamin, tHcys, cysteine, and As variables.

	tHcys	Cysteine	Plasma folate	Plasma cobalamin	Urinary creatinine
Water As (μg/L)					

Total	0.14	0.06	−0.11	0.08	
Females	0.08	0.08	−0.20	0.21	
Males	0.16	0.05	−0.04	−0.01	

uAs (μg/L)					

Total	0.01	−0.06	−0.22**	−0.03	
Females	−0.20	−0.16	−0.16	0.04	
Males	0.10	0.007	−0.25*	−0.04	

uAs/g creatinine					

Total	0.06	−0.10	−0.31***	−0.07	
Females	−0.08	−0.14	−0.36**	−0.14	
Males	0.15	−0.08	−0.23*	0.01	

Urinary %InAs					

Total	−0.003	−0.23**	−0.20*	−0.01	−0.31***
Females	−0.05	−0.16	−0.19	0.07	−0.31**
Males	0.04	−0.29**	−0.20	−0.09	−0.30**

Urinary %MMA					

Total	−0.18*,#	0.10	0.04	0.04	0.02
Females	−0.07	0.12	0.05	0.04	−0.04
Males	−0.27**	0.10	0.04	0.03	0.05

Urinary %DMA					

Total	0.13	0.11	0.12	−0.03	0.22**
Females	0.13	0.10	0.13	−0.12	0.27*
Males	0.12	0.14	0.11	0.07	0.19

* *p* < 0.05;

** *p* < 0.01;

*** *p* < 0.0001.

# Difference between females and males was statistically significant at *p* < 0.05.

**Table 3:** Mean levels of uAs metabolites in adults and children (mean ± SD).^a^

Metabolite	Adults CS (*n* = 293)^b^	10-year-old children (*n* = 201)^c^	6-year-old children (*n* = 165)^d^
%InAs	16.0 ± 8.9	12.8 ± 6.5	12.4 ± 5.3
%MMA	13.0 ± 5.4	9.3 ± 3.2	9.0 ± 3.6
%DMA	71.0 ± 10.0	77.9 ± 7.5	78.6 ± 6.3

CS, cross-sectional.

a Mean water As concentrations were 48 μg/L in the adults, 53 μg/L in the 10-year-old children, and 62 μg/L in the 6-year-old children.

b Previous cross-sectional study of Bangladeshi adults (Gamble et al. 2005b).

c Previous cross-sectional study of 10-year-old Bangladeshi children (Wasserman et al. 2004).

d Current study.
